# Supplementary material for: Controlling technical variation amongst 6693 patient microarrays of the randomized MINDACT trial
Source: Commun Biol. 2020 Jul 27;3:397. doi: 10.1038/s42003-020-1111-1 (PMC7385160; doi:10.1038/s42003-020-1111-1)
Supplement: Supplementary file 1 — Supplementary Information [file 42003_2020_1111_MOESM1_ESM.pdf]

Supplementary Figures

Supplementary Figure 1 – Missing probes

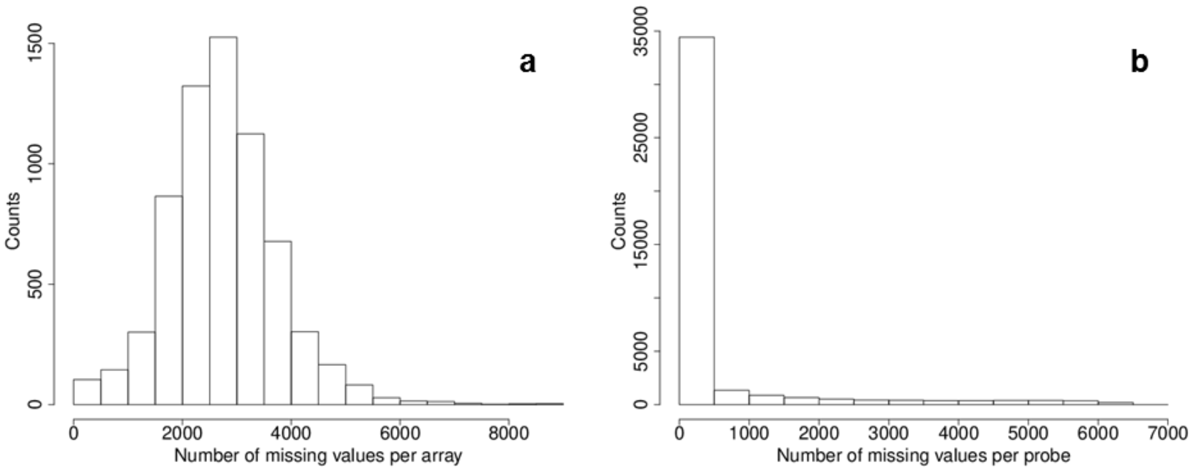

Panel a shows the distribution of the number of missing probes per patient array. The median number of missing values per array was 2701.5 (6.6%).

Panel b shows the distribution of the number of patient arrays in which probes were missing. Overall, 36,090 probes had at least one missing value across the 6,688 patient arrays. However, most probes were only missing from a few arrays (median = 5, or 0. 08%), and only a few probes were missing from a large number of arrays. Probes that were missing in more than 20% of any of the three groups of patient arrays defined by their reference RNA were filtered out for further analyses (n=5,182, or 13%; see Methods).

Supplementary Figure 2 – Principal component versus scanning time plots including control samples

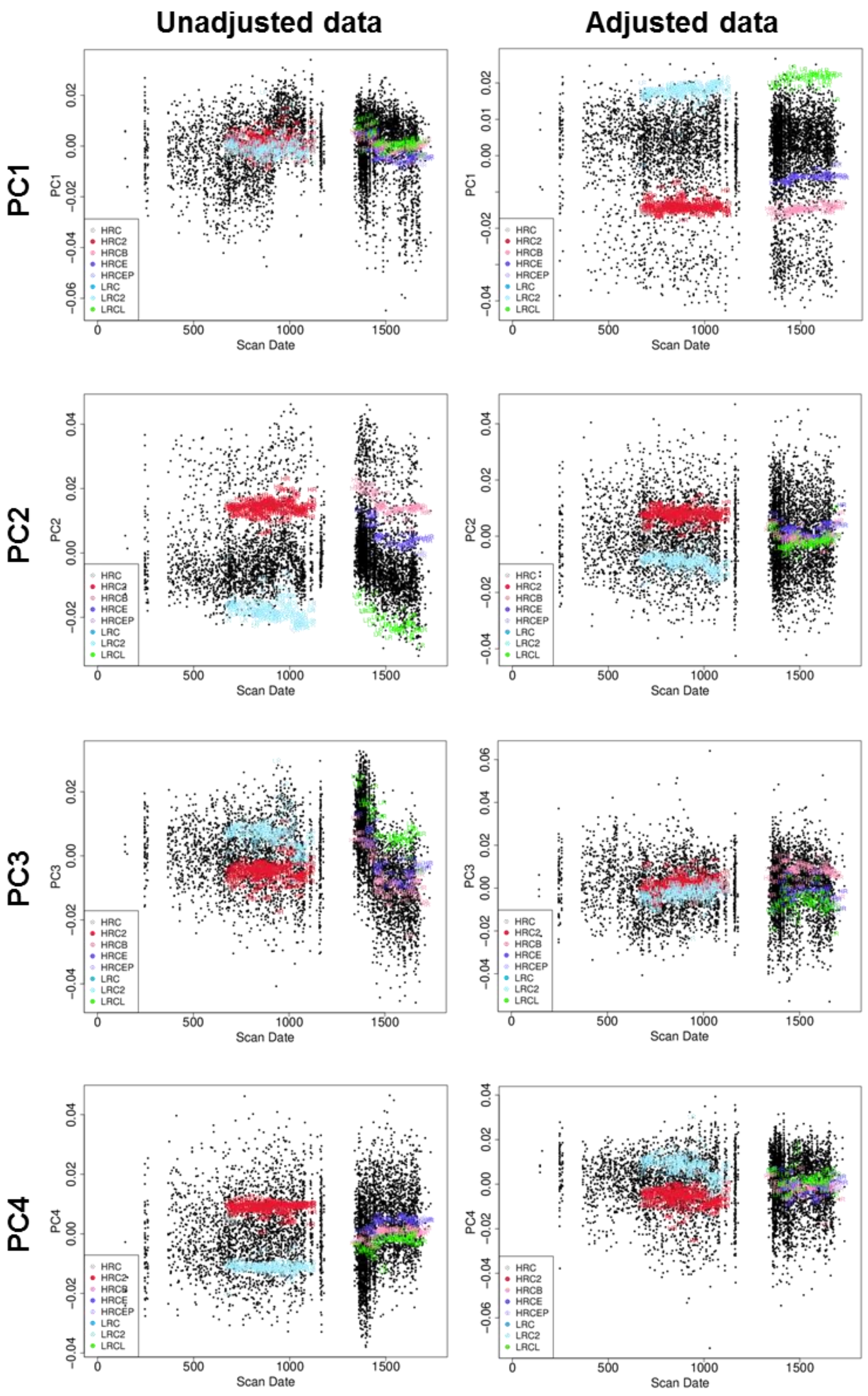

This figure represents the patient samples and control samples arrays by the top four principal components (PCs) against the time at which they were scanned, for the unadjusted and the adjusted dataset separately. The figure is organized in the same way as Figure 3, but also includes the 562 control arrays. During the time of the MINDACT trial eight different controls (LRC, LRC2 and LRCL,HRC, HRC2, HRCB, HRCE, and HRCEP) were used for

the purpose of assessment and control of technical variations over time (see Methods). We use these control samples to illustrate that the unadjusted data contains unwanted variation, and that the adjustment procedure removes this variation. The y-axis represents the projection of the samples onto each of the top four principal components for the unadjusted (left column) and the adjusted (right column) dataset. The x-axis represents the number of days between January 1st of 2007 and the scanning date of the samples instead of the isolation date that was shown in Figure 3 of the main manuscript. This is because control arrays are made using mixtures of samples that were isolated only once but were repeatedly hybridized and scanned at different times and used for quality control. The isolation of tumor samples took place at a constant rate over time, which can be seen in Figure 3 for there are no interruptions at the x-axis. The scan date of the arrays does show interruptions: they were caused by temporary unavailability of the full-transcriptome arrays and a temporary stop in the use of the full-transcriptome arrays while dealing with the manufacturer's change in RNA isolation reagent (see Methods).

These controls were hybridized multiple times and are plotted in this supplementary plot as well as in Figure 4. A caveat in the analysis of this figure is that control arrays are highly dependent, because a single control is used repeatedly, so if a small number of controls are outliers, it may look as if a large number of arrays had a different behavior. Nevertheless, we find it helpful to use these arrays to guide our interpretation of the PCs, keeping in mind that some observed effects may be the consequence of dependence among arrays which correspond to the same mixture.

Unadjusted data (left panels)

A first general comment regarding the left panels (unadjusted data) is that the PC projection of some controls change over the time. We emphasize that this has no implication on the stability of the MammaPrint score over time in the study -- this stability was established in a separate publication (Beumer et al., 2016). The MammaPrint score is a particular linear combination of 70 probes, which were specifically chosen to reflect the prognosis. By contrast, what is shown here in this Supplementary File are linear combinations intended to capture the largest proportion of variance across all arrays represented by all their probes. It is therefore not surprising that these combinations represent variations that also affect control arrays, and this by no means contradicts the stability of the MammaPrint score. Rather, this difference suggests that the MammaPrint score is not affected by the non-stationary variations that we observe along the first PCs.

The distributions of the PC1 projections of unadjusted patient arrays show fluctuations along scanning time. The two peaks in PC1 also correspond to larger values for control arrays. Each type of control samples contain the same RNA, so the fact that their projection along PC1 varies confirms that at least part of PC1 represents a technical factor, i.e., something which is not intrinsic to the biological sample and needs to be adjusted for. This variation also suggests that PC1 is related to more than isolation, e.g., labeling that is done repeatedly for a hybridization, since control arrays correspond to a single isolation. We also notice that the low-risk and high-risk controls are not well separated by PC1 in the unadjusted data, suggesting that PC1 in the unadjusted data has little to do with risk or prognosis.

The unadjusted PC2 clearly separates low-risk and high-risk controls, which reinforces its interpretation as a factor associated with ER/HER2.

The unadjusted control arrays are also strongly affected by the variations represented along PC3. The two peaks within the 6,688 unadjusted patient arrays also correspond to peaks of PC3 projections for the controls, which corroborates our hypothesis that PC3 is pulled by a technical effect unrelated to isolation.

Finally, the projections of unadjusted control arrays on PC4 have little variation over time, suggesting that PC4 is not associated with too much technical variation, or that this variation does not affect the controls. The controls are, however, affected by the small drop that occurs around day 1,300, right after the scan break, suggesting that this peak is indeed related to a technical effect.

Adjusted data (right panels)

PC projections of the unadjusted data are not one to one comparable with the PC projections of the adjusted data as PCs capture the largest proportion of variance across the samples in a dataset, and the structure of this variance is expected to be affected by the adjustment procedure.

The right panels represent the distribution of projections along the top four PCs computed on adjusted sample arrays, which shows that they are much more constant over time compared to the unadjusted data. Notably, the PC projections of control arrays are much more stable than before adjustment. We also notice that the low-risk and high-risk controls are now well separated by PC1 in the adjusted data, suggesting that PC1 in the adjusted data do associate with risk or prognosis. This data reinforces its interpretation as a factor associated with ER/HER2.

Beumer, I. *et al.* Equivalence of MammaPrint array types in clinical trials and diagnostics. *Breast Cancer Res. Treat.* **156,** 279–287 (2016).

Supplementary Figure 3 – Change in the time profiles of the principal components as a result of the adjustment procedure

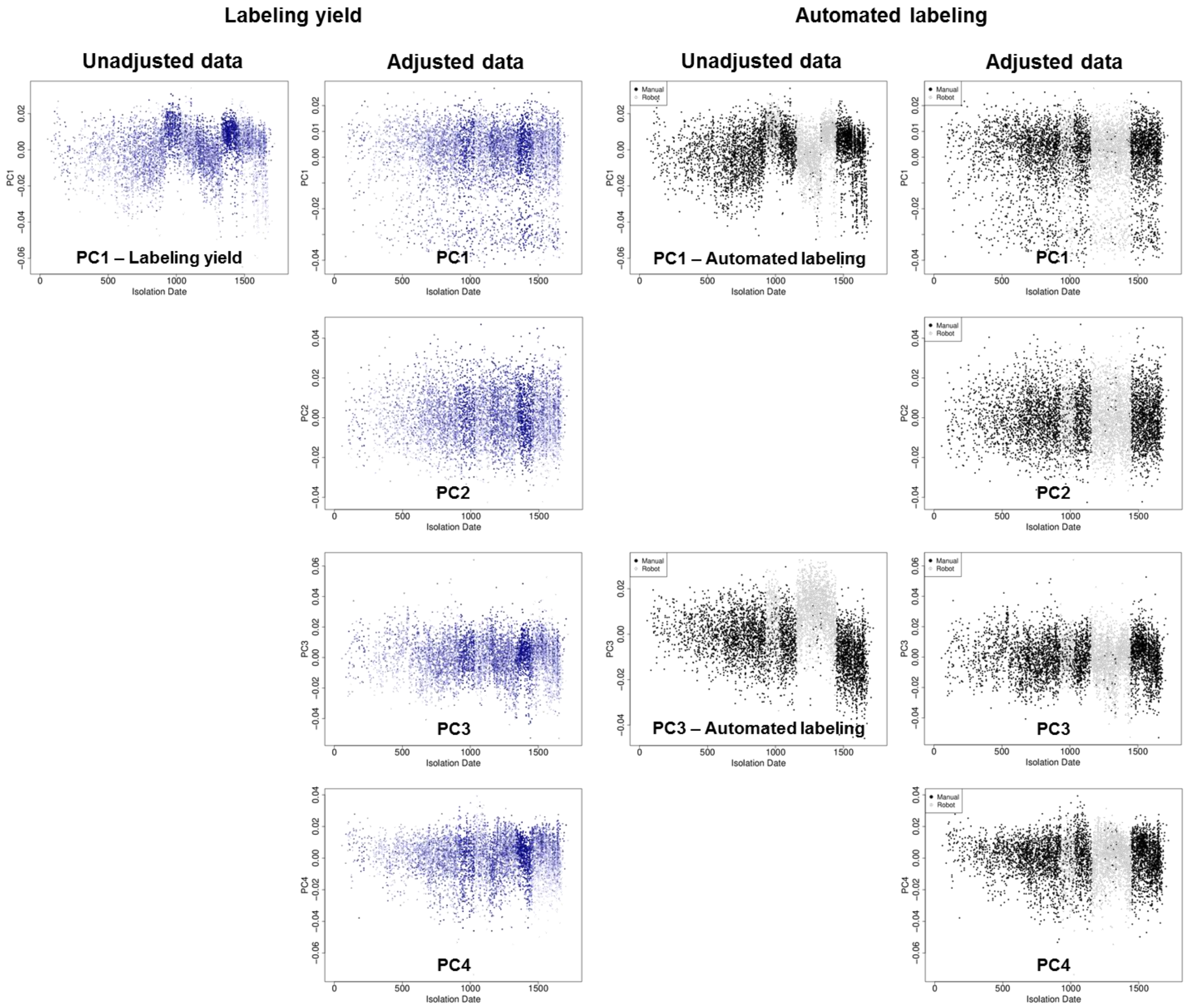

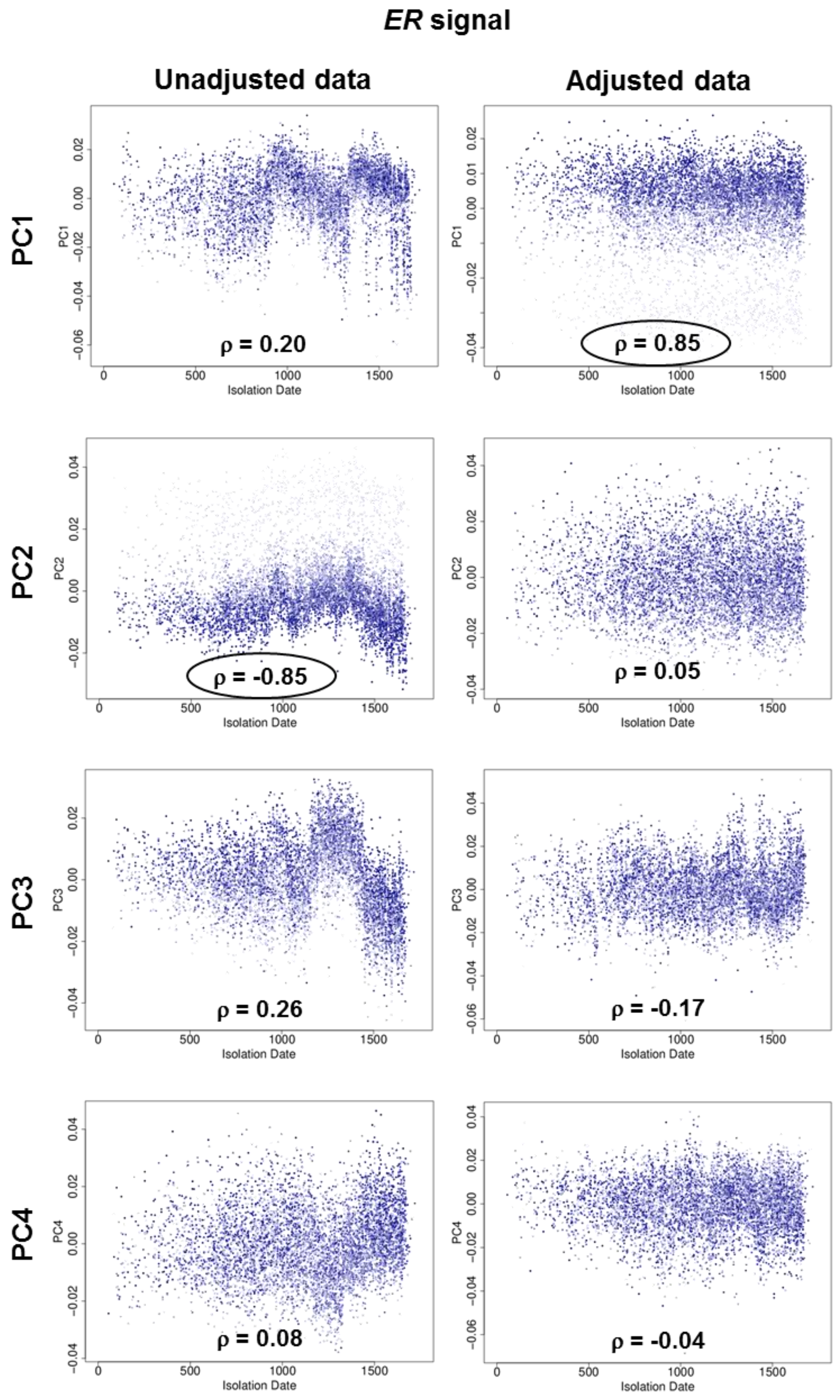
ER signal
Unadjusted data
Adjusted data
PC1
ρ = 0.20
ρ = 0.85
PC2
ρ = -0.85
ρ = 0.05
PC3
ρ = 0.26
ρ = -0.17
PC4
ρ = 0.08
ρ = -0.04
Isolation Date

We show the change in Principal Component (PC) vs time projections of technical and biological factors as result of the adjustment procedure in addition to Figure 5. Labeling yield, automated labeling and *ER* signal serve as examples to show that the variance explained by technical factors disappears (the distribution among samples becomes more stable over time), while variance explained by biological factors is visible in different PCs after data adjustment (also shown in Fig. 4). Principal component projections for the 6,688 patient samples after data adjustment were plotted against time. Each dot represents one patient sample. The x-axis represents the number of days between January 1st of 2007 and the isolation date of the sample. The y-axis represents the projection of the samples onto one of the top four principal components. For plots without color coding legends, the color intensity of the blue dots represents the value of the plotted signal, with high color intensities corresponding to high signals (see Methods). Abbreviations: *ER*, estrogen receptor; PC, Principal Component.

PC projections of the unadjusted data are not one to one comparable with the PC projections of the adjusted data: PCs capture the largest proportion of variance across the samples in a dataset, and the structure of this variance is expected to be affected by the adjustment procedure.

For direct comparison, we here show the plots for labeling yield and automated labeling (unadjusted data) from Figure 3 next to the representation of all top four PCs after data adjustment (adjusted data). When visually comparing the plots of the unadjusted and the adjusted data it is apparent that the fluctuations over time disappear. The technical factors labeling yield and automated labeling show associations with respectively PC1 and PC1/PC3 in the unadjusted data (as discussed in the Results section on step 2 and Fig. 3). The plots of the adjusted data in this Supplementary File visually confirm that such an association is not present after data adjustment in any of the top four PCs.

Additionally, we here show the plots for *ER* signal from Figure 3 (unadjusted data) and Figure 5 (adjusted data) next to the representation of all top four PCs of the unadjusted and adjusted data. The Pearson correlations of the *ER* signal with each PC are indicated at the bottom of each panel. For both the unadjusted and the adjusted data, a circle indicates the strongest association. The plots show that the association of *ER* signal shifted from PC2 to PC1 after data adjustment.

The plots of the unadjusted data show that the *ER* signal is strongest on PC2, but it is also present on PC1 and PC3. A blue color intensity gradient along the indicated PC is indicative for such an association. Using a naive adjustment aimed at removing all the signal along PC1 and PC3 (the 2 PCs carrying the technical effects) could therefore erase some of the *ER* signal. This is exactly why we chose to use the RUV method instead (see Methods and Discussion section).

Supplementary Figure 4 – PC projections of the unadjusted and adjusted data color-coded for the main biological signals studied in the manuscript

For the sake of comprehensibility, here we show together the projections of the 6688 samples over the first four principal components, color-coded for the five main biological signals discussed in the manuscript (BRCA, CLDN, ER, HER2, TP53). The first table shows these projections for the unadjusted dataset, the second table for the adjusted dataset. These tables are an extended version of the Figure 4 of the main manuscript. Alternatively, associations between these PC projections and biological signals are provided in Supplementary Data 1.

Unadjusted data

BRCA

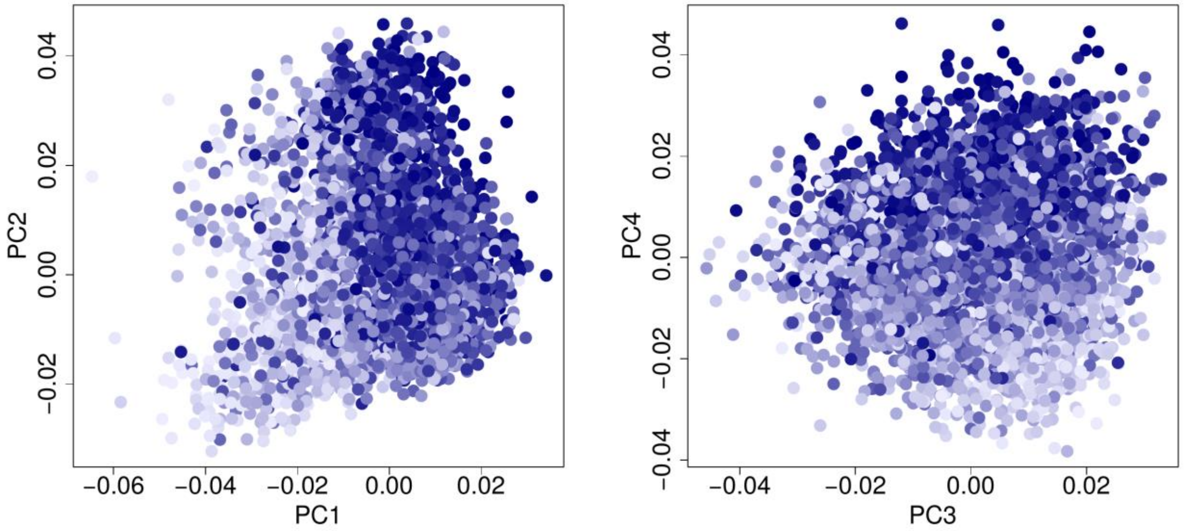

CLDN

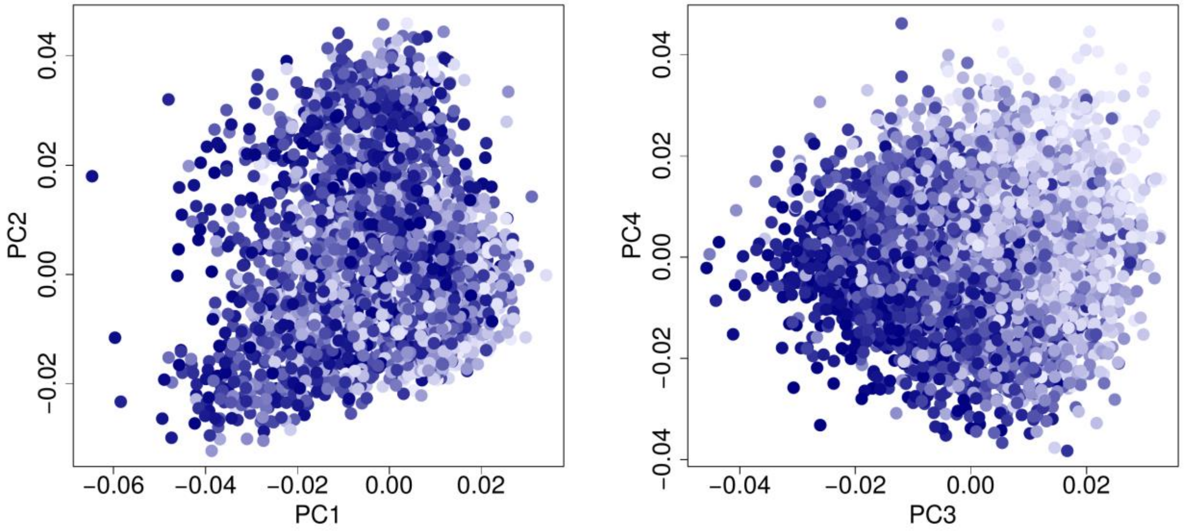

ER

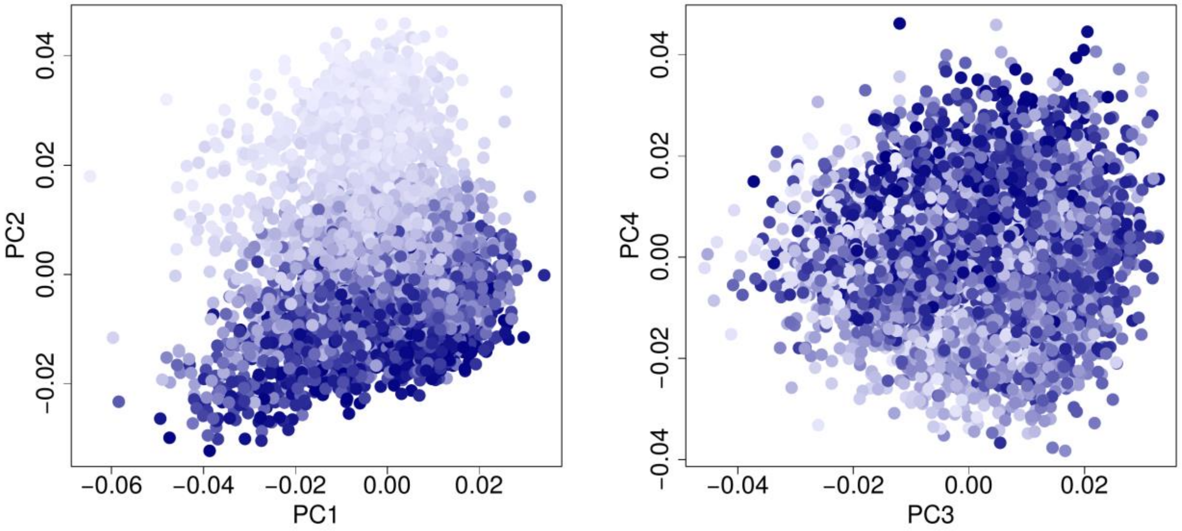
PC2
PC1
−0.06 −0.04 −0.02 0.00 0.02
−0.02 0.00 0.02 0.04
PC4
PC3
−0.04 −0.02 0.00 0.02
−0.04 −0.02 0.00 0.02 0.04

HER2

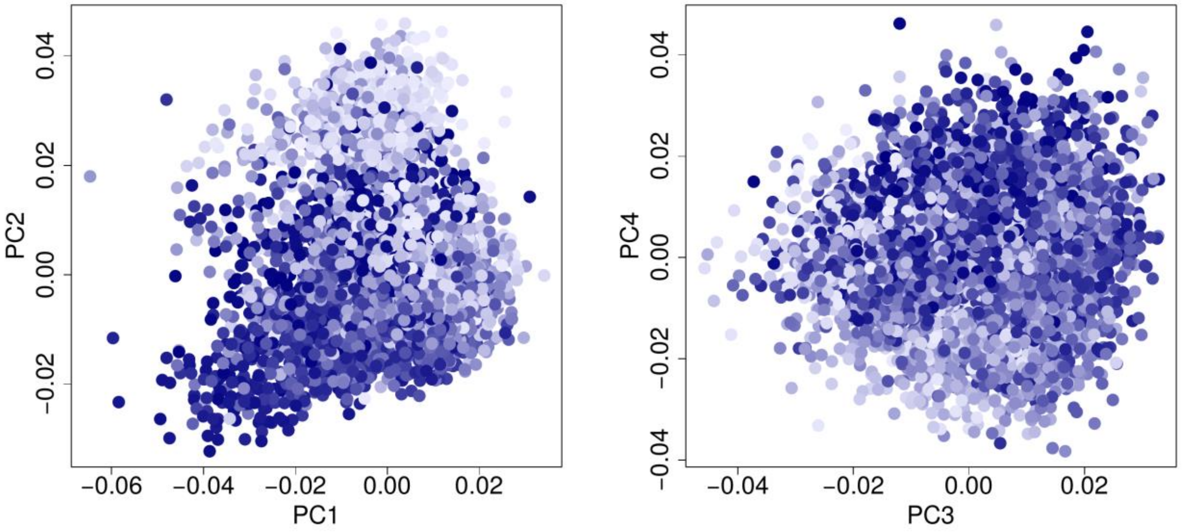
PC2
PC1
−0.06 −0.04 −0.02 0.00 0.02
−0.02 0.00 0.02 0.04
PC4
PC3
−0.04 −0.02 0.00 0.02
−0.04 −0.02 0.00 0.02 0.04

TP53

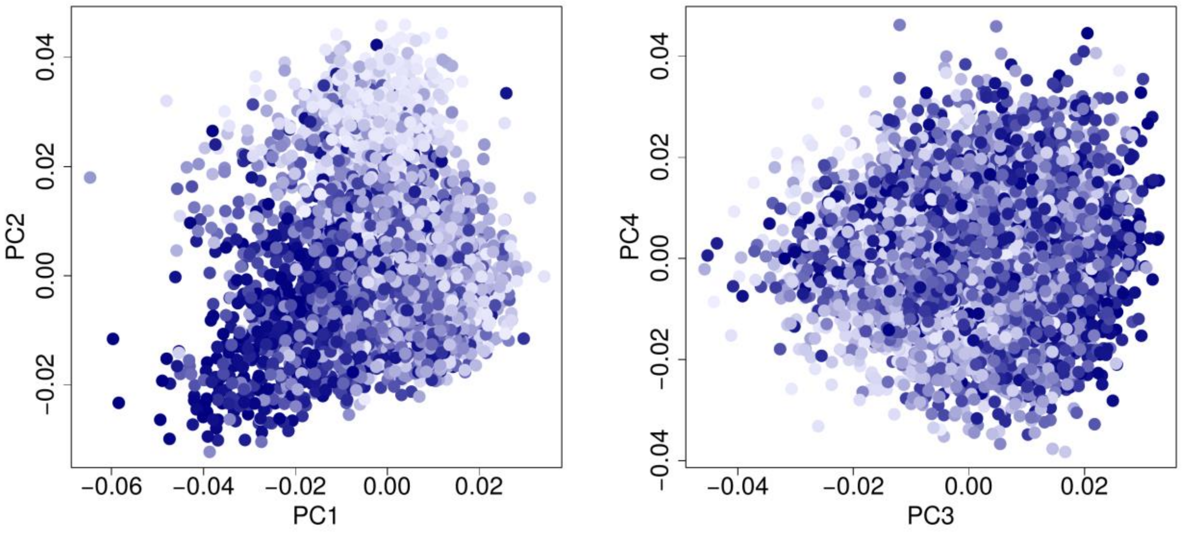

Adjusted data

BRCA

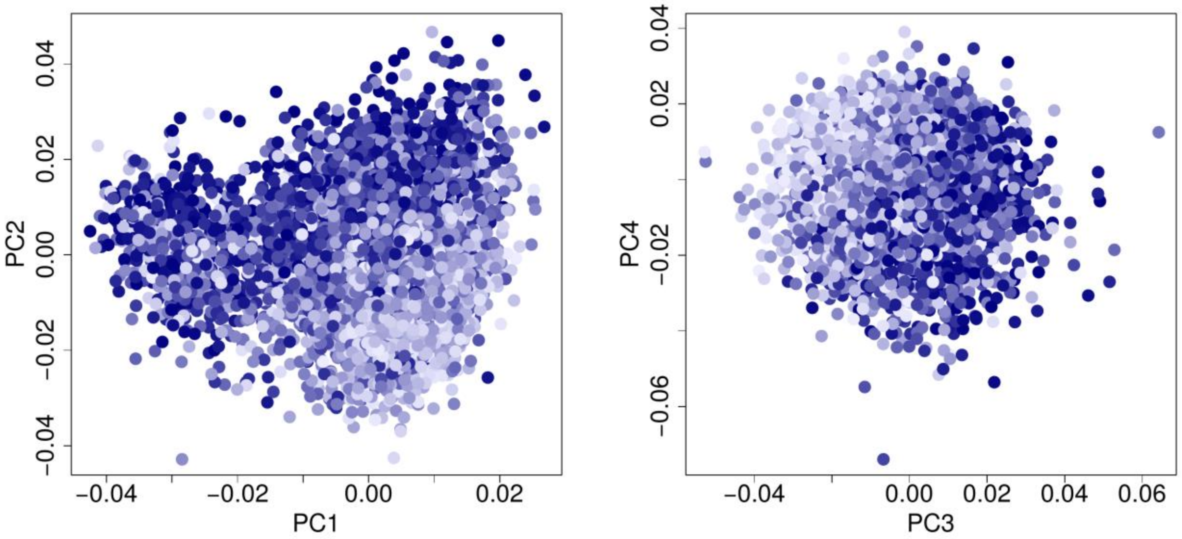

CLDN

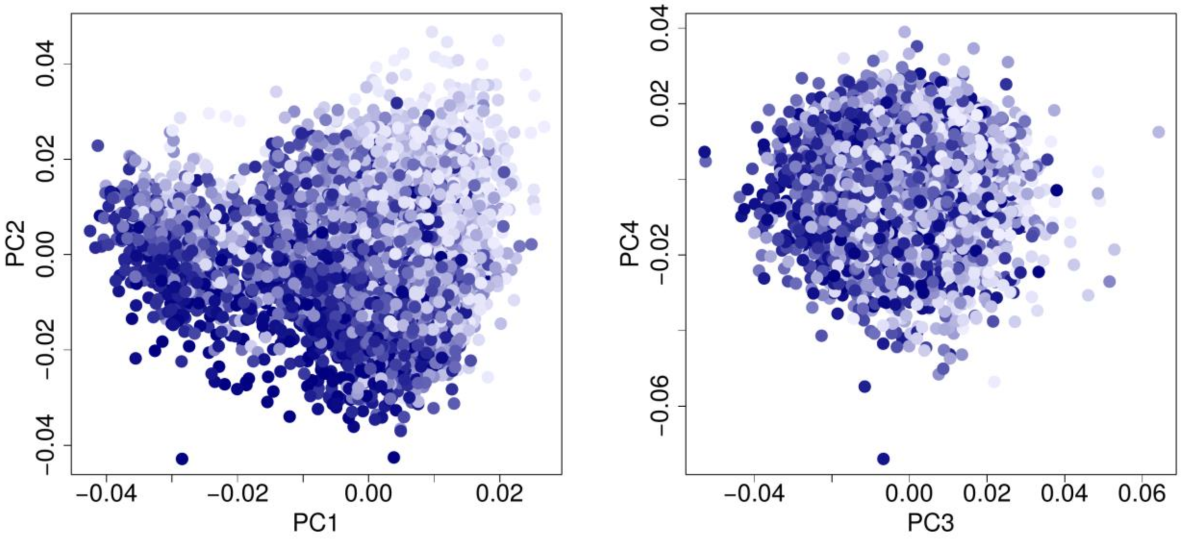
PC2
0.04
0.02
0.00
−0.02
−0.04
−0.04
−0.02
0.00
0.02
PC1
PC4
0.04
0.02
−0.02
−0.06
−0.04
0.00
0.02
0.04
0.06
PC3

ER

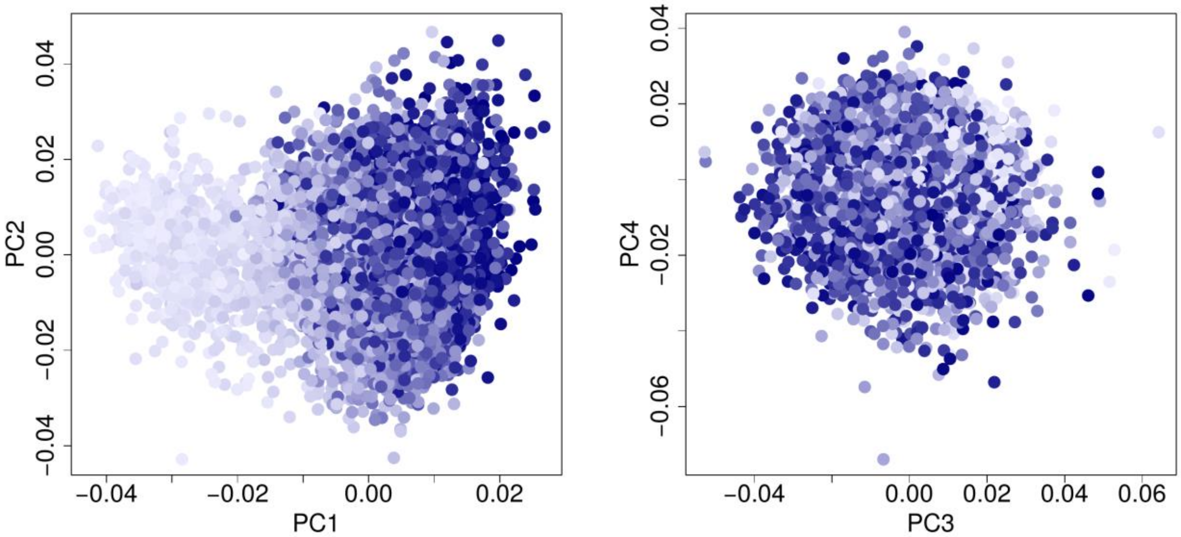
PC2
0.04
0.02
0.00
−0.02
−0.04
−0.04
−0.02
0.00
0.02
PC1
PC4
0.04
0.02
−0.02
−0.06
−0.04
0.00
0.02
0.04
0.06
PC3

HER2

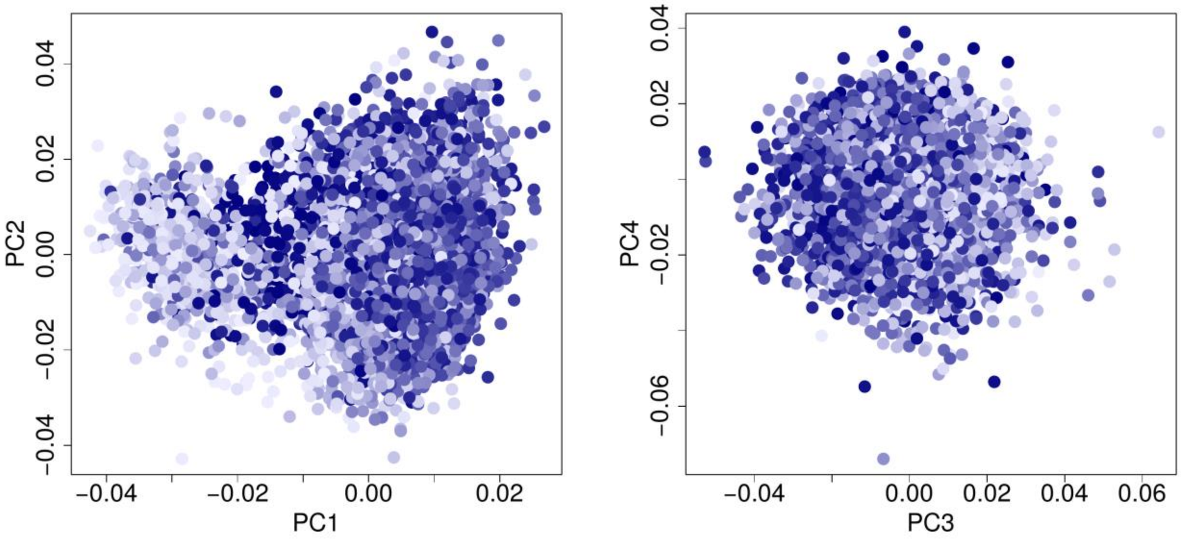
PC2
PC1
PC4
PC3
0.04
0.02
0.00
−0.02
−0.04
−0.06
0.06

TP53

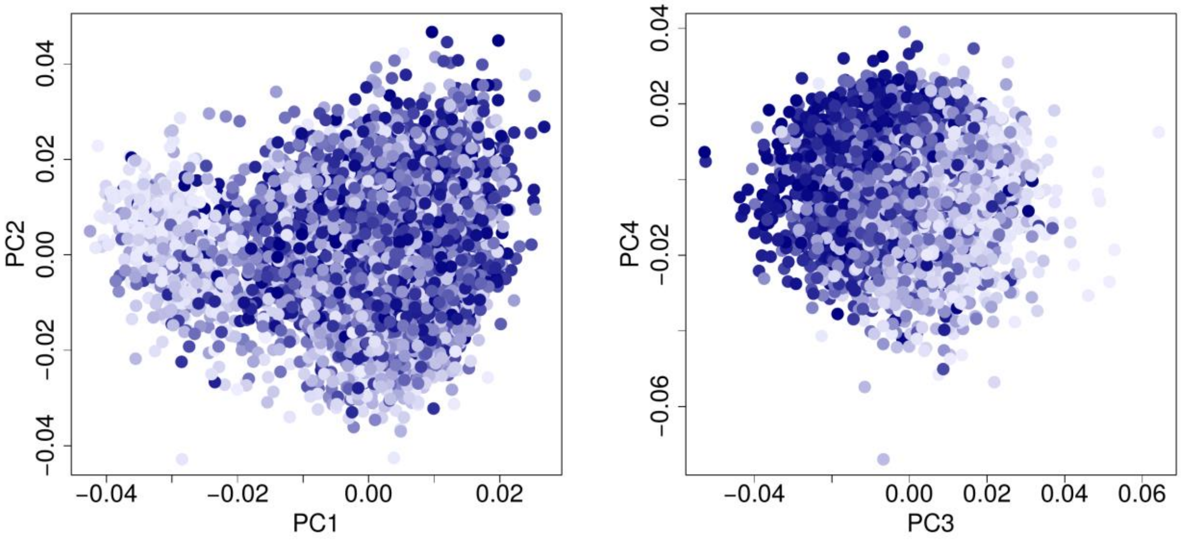
PC2
PC1
PC4
PC3
0.04
0.02
0.00
−0.02
−0.04
−0.06
0.06

Supplementary Tables

Supplementary Table 1 – List of investigated technical information and changes

Location	Lab location – Boolean factor identifying the lab location
Isolation	Tumor cell percentage – percentage of tumor cells in the sample<br>RNA isolation date – date of RNA isolation<br>Isolation buffer – isolation reagent used<br>Automated isolation – Boolean factor identifying manually versus automated DNAse treatment during isolation<br>Isolation equipment – identifying if a Polytron or Tissue Lyser was used for homogenization<br>Isolation technician – identifying the technician that performed the isolation procedure<br>RIN – RNA integrity number (RNA quality assessed using BioAnalyzer from Agilent)<br>BA ratio – Quality indicator of RNA quality assessed by the BioAnalyzer lab instrument<br>ND conc – Nanodrop concentration
Labeling	Labeling date – date of labeling<br>Automated labeling – Boolean factor identifying manually versus automated labeling<br>Labeling technician – identifying the technician that performed the labeling procedure<br>Labeling incorporation sample – Level of incorporation of the Cyanine 3-CTP dye<br>Labeling yield sample – Total Amount of incorporated Cyanine 3-CTP<br>Labeling incorporation reference – Level of incorporation of the Cyanine 5-CTP dye<br>Labeling yield reference – Total Amount of incorporated Cyanine 5-CTP
Hybridization	Hybridization technician – identifying the technician that performed the array hybridization<br>Reference ID – identifying the reference RNA used<br>Sub array – identifying the location of the sample on the microarray
Scanning	Scan date – date of scanning<br>Scanner – factor identifying the scanner used

.

Supplementary Notes, Discussion, Methods

Supplementary Note 1 – Analysis of sample correlations

To assess whether any of the reported arrays may correspond to the same sample, we studied pairwise Pearson correlations among all pairs of arrays, each array being represented by a vector with one entry per measured probe. These correlations were computed across the probes that were not missing in any of the arrays (n= 4,703 probes) to avoid potential introduction of artifacts from the missing value imputation procedure. Results of the pairwise correlation analysis for the first 1,600 samples identified four sample pairs (eight distinct arrays) with a Pearson correlation ρ>0. 9. To determine if this high correlation was caused by an actual duplication, available RNA was reprocessed for those five out of the eight samples that had enough RNA available. Subsequently, we compared their MammaPrint indices to the original indices. Results indicate that two samples, for which the 14-character labeling ID differed only for one digit, were subject to sample mix-ups preceding the microarray hybridization step, and in these cases the risk calls changed. The MammaPrint indices of the other three reprocessed samples showed to be highly reproducible, indicating that the same sample was processed. The dataset available through the EORTC (https://www.eortc.org/data-sharing/) contains the reprocessed versions of these two samples. After reprocessing, the correlations dropped to 0.17 and 0.4 respectively.

Results of the pairwise correlation analysis over the complete set of 6,688 arrays identified 6,368 pairs with a correlation >0.9. The figure below shows the 10,000 largest correlations between pairs or arrays scanned less than 21 days apart. To reduce the size of this set, we restricted ourselves to 15 pairs of arrays that were scanned less than 21 days apart and that showed a correlation larger than or equal to 0.95 with at least one other sample (up to five). Thirteen samples that satisfied these criteria and for which enough RNA was available were reprocessed. Results indicated that the MammaPrint indices of all reprocessed samples were consistent with their original versions and the intra-pair sample correlation remained high. This consistency suggests that high correlations (of those probes without missing values) are not necessarily indicative for two samples being identical.

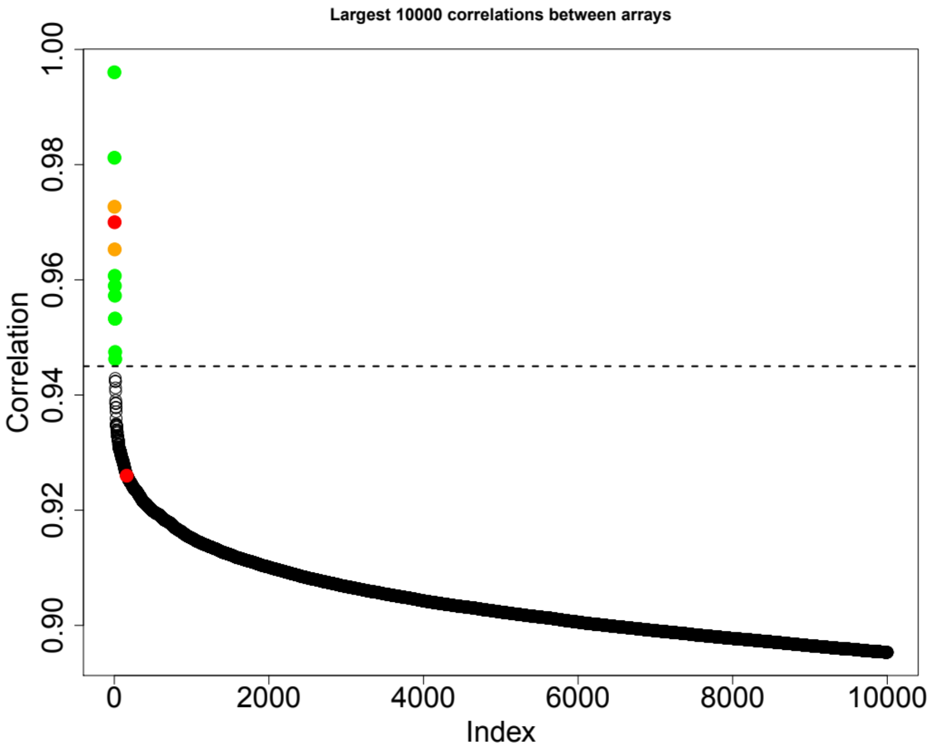

The figure shows the 10,000 largest correlations between pairs or arrays scanned less than 21 days apart. The red dots are the two pairs described above (before reprocessing and replacement in the EORTC dataset) identified in the first 1600 samples. The orange dots represent the pairs, for which at least one sample could not be reprocessed. The green dots are the other pairs above the threshold.

Supplementary Note 2 – Alternative adjustments

Adjustment without using the isolation reagent or labeling robot information

Applying RUV without explicitly adjusting for the labeling robot or isolation reagent, it is possible to decrease the association of the first four PCs with technical factors but Supplementary File 7 shows that it requires decreasing the nu.coeff parameter (which controls how much variance is removed by the adjustment (see Methods and Supl File 7) to the point where the correlation between PC1 and the TargetPrint-*ER* scores goes down to 0.2. This unsatisfactory trade-off is likely to result from two points. First, the technical factors explain a large proportion of the overall variance, so correcting for their effect requires an adjustment of large magnitude (small nu.coeff parameter). RUV uses negative control genes to estimate the unwanted variation factors it adjusts for, so if the expression of these negative control genes has a non-zero correlation with TargetPrint-*ER*, the large adjustment automatically decreases the TargetPrint-*ER* signal. Second, we are using the 1,000 genes with lowest inter-quartile range (IQR) as negative controls, and these are likely to have a non-zero correlation with TargetPrint-*ER*.

Principal component regression

Figure 4 of the manuscript shows that the grouping of tumors by *ER*+/*ER*- status, which is the main expected biological signal is associated with PC2 of the unadjusted data and moves to PC1 after adjustment. The technical variation associated with labeling present on PC3 before adjustment also disappears after adjustment. A simpler method to obtain this result would be to project the arrays onto the subspace orthogonal to PC1 and PC3, i.e., to remove all variation along PC1 and PC3 in the data. The new PC1 computed on the data after this transformation would be as a direct consequence equal to the PC2 of the unadjusted data, corresponding to the ER signal. However as discussed in the manuscript, each PC is typically associated with several signals: on the unadjusted data, PC1 is also associated with *ER* and *BRCA* while the distribution of PC2 projections fluctuate over time (Fig. 3, Supplementary File 4), suggesting it is also associated with some technical variation. Figure 5 and Supplementary File 4 show that our adjustment does more than just move the former PC2 to PC1, as the distribution of PC1 projections computed on the adjusted data is more stationary than the distribution of PC2 projections computed before adjustment. More generally, we expect our adjustment to remove other subtler technical variation orthogonal to PC1 and PC3.

Supplementary Note 3 – Effect of the adjustment parameter nu.coeff on the adjustment

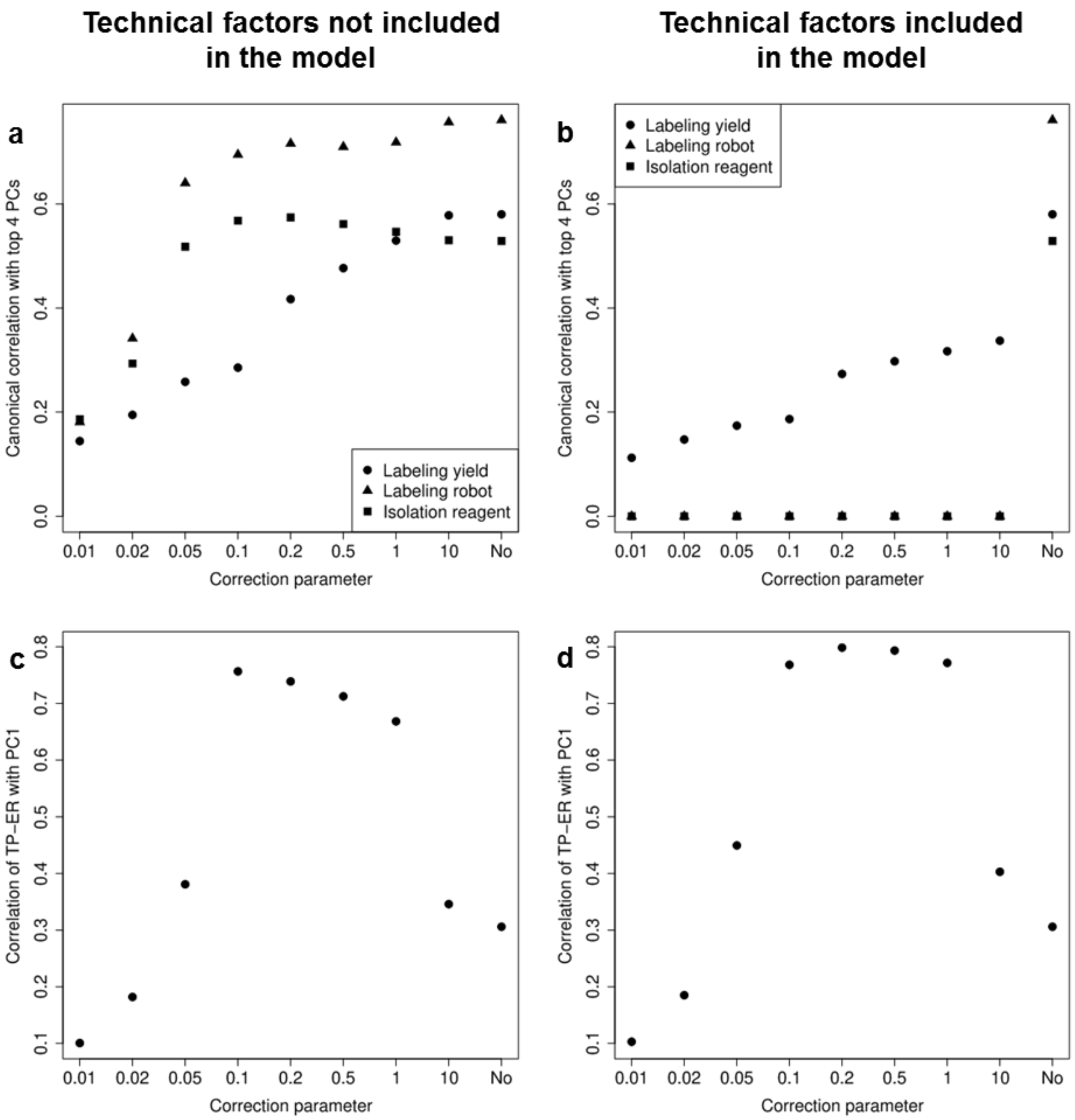

The adjustment method which we use in this paper can be tuned using a "correction parameter" called "nu.coeff", which controls how much variance is removed by the adjustment (see Methods). Larger values of nu.coeff lead to removing less variance, yielding a conservative approach which is less likely to lose signals of interest, at the cost of potentially keeping more unwanted variation (see (Jacob *et al*., 2016) for more details). In order to choose the value of this parameter, we consider both positive and negative controls. Our positive control is the TargetPrint-*ER* (TP) score of each sample. This score is computed by reading one particular *ER* probe on the array, and has a concordance larger than 95% with the immunochemistry *ER* status of the samples after binarization (Roepman *et al*., 2009). It is desirable that after any adjustment, the first PC is highly correlated

with the original TP readout, since the main expected signal in a breast cancer dataset is a clustering by *ER*+/*ER*- molecular subtypes. On the other hand, we know from our analysis of the unadjusted data (see step 3 of the Results section) that gene expression is affected by labeling robot, labeling yield and isolation reagent. A good adjustment method should remove as much as possible of these effects.

Panels a and c of the figure show the effect of nu.coeff on a plain RUV adjustment, which unlike the adjustment used in this manuscript does not take into account known technical effects (Section “Adjustment without using the isolation reagent, labeling robot or labeling yield information” in Supplementary File 6). In panel a, we can see that smaller values of the nu.coeff parameter lead to smaller associations between unwanted variation factors and the first principal components, but that this association remains large unless nu.coeff is equal to 0.02 or smaller. At the same time, panel c shows that smaller values of nu.coeff also make the *ER* probe less correlated with the TP-*ER* score. When nu.coeff is equal or smaller than 0.02, the bottom panel shows that the association between the first PC and TP-*ER* becomes lower than 0.2.

Panels b and d of the figure show the effect of nu.coeff on the hybrid adjustment that we use in the main manuscript: regressing out the isolation reagent and labeling robot factors while applying RUV (see Methods). By construction, the adjustment removes any association between the measured gene expression and isolation reagent or labeling robot. The association with labeling yield which is not explicitly adjusted for decreases when nu.coeff decreases, as in the previous case. The correlation between TP-*ER* score and PC1 reaches a maximum for nu.coeff=0.2, but any value between 0.1 and 1 would give very similar results.

Jacob, L., Gagnon-Bartsch, J. A. & Speed, T. P. Correcting gene expression data when neither the unwanted variation nor the factor of interest are observed. *Biostatistics* **17,** 16–28 (2016).

Roepman, P. *et al.* Microarray-based determination of estrogen receptor, progesterone receptor, and HER2 receptor status in breast cancer. *Clin. Cancer Res.* **15,** 7003–7011 (2009).
