# Supplementary material for: Controlling technical variation amongst 6693 patient microarrays of the randomized MINDACT trial
Source: Commun Biol. 2020 Jul 27;3:397. doi: 10.1038/s42003-020-1111-1 (PMC7385160; doi:10.1038/s42003-020-1111-1)
Supplement: Supplementary file 4 — Description of Additional Supplementary Files [file 42003_2020_1111_MOESM4_ESM.pdf]

Supplementary code 1 – This file contains the code that reproduces the analysis of the 6688 patient arrays as well as their adjustment for technical variation. It generates the adjusted data matrix available from the EORTC and figures.

Supplementary Data 1 – Associations of technical and biological factors with the top four PCs
We provide the association of all reported technical and biological factors with the top four principal components computed on the unadjusted and adjusted data. The factors are organized in three tables: biological factors (which are all continuous), continuous technical factors and categorical technical factors. Within each table, factors are sorted by decreasing association with the top four PCs before adjustment.

The association between each single PC and continuous factors are measured using Pearson correlation. Global associations of factors with the top four PCs are measured using canonical correlations. All associations of PCs with categorical factors are also computed using canonical correlations between the PC or PCs and a one-hot encoding matrix of the categorical factor. Each row of the matrix corresponds to an array, each column to a possible level of the factor, with values 1 at rows corresponding to arrays having this level and 0 elsewhere.

Of note, the ordering and comparison of canonical correlations with categorical factors are only indicative, since canonical correlation mechanically increases with the number of categories in the factor.
