# Supplementary material for: Controlling technical variation amongst 6693 patient microarrays of the randomized MINDACT trial
Source: Commun Biol. 2020 Jul 27;3:397. doi: 10.1038/s42003-020-1111-1 (PMC7385160; doi:10.1038/s42003-020-1111-1)
Supplement: Supplementary file 5 — Reporting Summary [file 42003_2020_1111_MOESM5_ESM.pdf]

nature research

Corresponding author(s): LJ van 'tVeer and AM Glas

Last updated by author(s): Jun 15, 2020

Reporting Summary

Nature Research wishes to improve the reproducibility of the work that we publish. This form provides structure for consistency and transparency in reporting. For further information on Nature Research policies, see Authors & Referees and the Editorial Policy Checklist.

Statistics

For all statistical analyses, confirm that the following items are present in the figure legend, table legend, main text, or Methods section.

n/a	Confirmed	
☐	☒	The exact sample size (*n*) for each experimental group/condition, given as a discrete number and unit of measurement
☐	☒	A statement on whether measurements were taken from distinct samples or whether the same sample was measured repeatedly
☒	☐	The statistical test(s) used AND whether they are one- or two-sided<br>*Only common tests should be described solely by name; describe more complex techniques in the Methods section.*
☐	☒	A description of all covariates tested
☒	☐	A description of any assumptions or corrections, such as tests of normality and adjustment for multiple comparisons
☐	☒	A full description of the statistical parameters including central tendency (e.g. means) or other basic estimates (e.g. regression coefficient) AND variation (e.g. standard deviation) or associated estimates of uncertainty (e.g. confidence intervals)
☒	☐	For null hypothesis testing, the test statistic (e.g. *F*, *t*, *r*) with confidence intervals, effect sizes, degrees of freedom and *P* value noted<br>*Give P values as exact values whenever suitable.*
☒	☐	For Bayesian analysis, information on the choice of priors and Markov chain Monte Carlo settings
☐	☒	For hierarchical and complex designs, identification of the appropriate level for tests and full reporting of outcomes
☐	☒	Estimates of effect sizes (e.g. Cohen's *d*, Pearson's *r*), indicating how they were calculated

Our web collection on statistics for biologists contains articles on many of the points above.

Software and code

Policy information about availability of computer code

Data collection	No software was used for collection of the data. Existing data from full genome microarray experiments was used.
Data analysis	For data analysis R (version 3.4.4, https://www.r-project.org/) was used. Code to reproduce the plots in the manuscript is deposited at the EORTC and available along site the manuscript as supplementary materialand through request from the EORTC ((https://www.eortc.org/data-sharing/).

For manuscripts utilizing custom algorithms or software that are central to the research but not yet described in published literature, software must be made available to editors/reviewers. We strongly encourage code deposition in a community repository (e.g. GitHub). See the Nature Research guidelines for submitting code & software for further information.

Data

Policy information about availability of data

All manuscripts must include a data availability statement. This statement should provide the following information, where applicable:

- Accession codes, unique identifiers, or web links for publicly available datasets
- A list of figures that have associated raw data
- A description of any restrictions on data availability

The dataset is available (unadjusted and adjusted) by submitting proposals to the EORTC for access to the gene expression data (https://www.eortc.org/data-sharing/).

Field-specific reporting

Please select the one below that is the best fit for your research. If you are not sure, read the appropriate sections before making your selection.

☒ Life sciences ☐ Behavioural & social sciences ☐ Ecological, evolutionary & environmental sciences

For a reference copy of the document with all sections, see nature.com/documents/nr-reporting-summary-flat.pdf

Life sciences study design

All studies must disclose on these points even when the disclosure is negative.

Sample size	In total 6,693 samples were used, all samples that were enrolled in The MINDACT randomized breast cancer trial
Data exclusions	Data from 5 samples were excluded based on poor quality
Replication	Experimental findings were reproduced by running the code at an independent institution where the data is stored and will be made available
Randomization	Randomization of samples is not applicable in this study as we used only existing expression data, no clinical data is used.
Blinding	Blinding is not relevant as no clinical data was used in the study

Reporting for specific materials, systems and methods

We require information from authors about some types of materials, experimental systems and methods used in many studies. Here, indicate whether each material, system or method listed is relevant to your study. If you are not sure if a list item applies to your research, read the appropriate section before selecting a response.

Materials & experimental systems

n/a	Involved in the study	
☒	☐	Antibodies
☒	☐	Eukaryotic cell lines
☒	☐	Palaeontology
☒	☐	Animals and other organisms
☐	☒	Human research participants
☒	☐	Clinical data

Methods

n/a	Involved in the study	
☒	☐	ChIP-seq
☒	☐	Flow cytometry
☒	☐	MRI-based neuroimaging

Human research participants

Policy information about studies involving human research participants

Population characteristics	The MINDACT randomized trial enrolled 6,693 breast cancer patients representative for stage I-II primary diagnosis.
Recruitment	Gene expression data generated from patients enrolled in the mindact study was used.
Ethics oversight	The protocol review committee of the European Organization for Research and Treatment of Cancer (EORTC) and the ethics committee at each participating site approved the study.

Note that full information on the approval of the study protocol must also be provided in the manuscript.
